# Normal variation of clinical mobility of the mandibular symphysis in dogs

**DOI:** 10.3389/fvets.2023.1260451

**Published:** 2023-11-16

**Authors:** Sergio Minei, Edoardo Auriemma, Serena Bonacini, Michael S. Kent, Margherita Gracis

**Affiliations:** ^1^Department of Dentistry, Oral, and Maxillofacial Surgery, Istituto Veterinario di Novara AniCura, Granozzo con Monticello, Novara, Italy; ^2^Department of Dentistry, Oral, and Maxillofacial Surgery, Clinica Veterinaria San Siro AniCura, Milan, Italy; ^3^Department of Diagnostic Imaging, Istituto Veterinario di Novara AniCura, Granozzo con Monticello, Novara, Italy; ^4^Dentistry, Oral, and Maxillofacial Surgery Service, School of Veterinary Medicine, William R. Pritchard Veterinary Medical Teaching Hospital, University of California, Davis, Davis, CA, United States; ^5^Department of Surgical and Radiological Sciences, Center for Companion Animal Health, School of Veterinary Medicine, University of California, Davis, Davis, CA, United States

**Keywords:** mandible, symphysis, mandibular symphyseal morphology, mandibular symphyseal mobility, intraoral radiography, dogs

## Abstract

**Introduction:**

The primary objective of this retrospective study was to document the normal variation of clinical mobility of the mandibular symphysis in dogs, and evaluate possible associations with breed, bodyweight, age, sex, and skull morphology. Secondarily, the radiographic appearance of the mandibular symphysis and possible associations with the analyzed data were also evaluated.

**Methods:**

Medical records of dogs that underwent anesthetic procedures for maxillofacial, oral and dental evaluation from April 2015 to December 2021 were included.

**Results:**

567 dogs of 95 different breeds were included, with a total of 695 evaluations. Body weight ranged from 0.8 kg to 79 kg (median 14.4 kg) and age from 3 months to 16 years and 4 months (median 6 years and 9 months). Clinical mobility was evaluated under general anesthesia using a 0 to 3 scale, in lateromedial (LM) and dorsoventral (DV) directions. The symphysis was radiographically classified as being fused or open. The open symphyses were further radiographically divided in having parallel or divergent margins. At the time of the first evaluation DV mobility was 0 in 551 cases (97.2%) and 1 in 16 cases (2.8%). LM mobility was 0 in 401 cases (70.7%), 1 in 148 cases (26.1%) and 2 in 18 cases (3.2%). There was not a significant change in mobility over time for cases examined more than once (*P*= 0.76). All cases had an intraoral radiographic examination. 83.8% of the radiographs were included in the statistical analysis. Two symphyses (0.4%) were classified as fused and 473 (99.6%) as open, 355 (74.7%) having divergent margins and 118 (24.8%) parallel margins. Logistic regression models exploring factors that affected DV and LM mobility were statistically significant (*P* < 0.0001; *P* < 0.0001), with an increase in LM mobility predicting an increase in DV mobility, and vice versa. An increase in age and in bodyweight was associated with a decrease in mobility. There was no statistical difference in clinical mobility across specific breeds or sexes. Increased probability of a divergent symphysis and increased DV mobility was found to be associated with a brachycephalic conformation. The increase in LM mobility was comparatively higher in small brachycephalic breeds compared with larger brachycephalic breed.

**Discussion:**

The majority of the cases showed little to no mobility of the mandibular symphysis and radiographically bony fusion can be rarely seen.

## 1 Introduction

The mandibular symphysis connects right and left mandibles at the rostral portion of the mandibular body, the pars incisiva (1). In animals the morphology of the mandibular symphysis varies significantly, as it may appear as a synchondrosis or amphiarthrosis (i.e., a cartilaginous joint, with smooth, opposing symphyseal plates connected by fibrocartilage and ligaments), a synarthrosis (i.e., a fibrous joint, with symphyseal plates with interlocking irregularities and variable amounts of dense or calcified connective tissues) or a synostosis (i.e., a fully ossified, or fused, joint), with various degrees of mobility and flexibility (2–7). All types of symphyseal joints have been observed in carnivores, with great variation within different families, genera and even species (3). In dogs in particular, the symphysis is described as a synchondrosis with a high degree of mobility (3, 8). It has been speculated that a flexible symphyseal joint can better absorb occlusal shocks, and reduce the risk for fracturing carnassial teeth, allowing adaptation to different diets and to a unilateral masticatory mechanism of action, typical of dogs (2, 3, 9–13). However, the normal degree of mobility that is to be expected in clinical canine patients is unknown. Also, radiographically, the symphysis has been described as a radiolucent structure (14–16), but little detail can be found in the literature. The aim of this study was therefore to assess the clinical degree of mobility of the mandibular symphysis in dogs, and evaluate possible breed, bodyweight, age, sex and skull morphology differences. The secondary aim was to correlate the clinical mobility and the other collected data to symphyseal radiographic appearance and shape.

## 2 Materials and methods

All animals included in the study were client-owned dogs anesthetized between April 2015 and December 2021 because of oral or maxillofacial problems and procedures. Data collected for each animal included signalment (i.e., age, sex, neutering status, breed, bodyweight, skull morphology as brachycephalic or non-brachycephalic), clinical symphyseal mobility, radiopacity and radiographic shape of the mandibular symphysis. Statistical analysis on data for the first evaluation was performed on the study population as a whole. Bodyweight was also evaluated based on the following categorical groups [subgroup 1 (≤5 kg), subgroup 2 (5.1–10 kg), subgroup 3 (10.1–25 kg), and subgroup 4 (≥25.1 kg)]. Age was also evaluated by classifying dogs as either immature dogs ( ≤15 months of age) or mature dogs (≥15.1 months of age) at the time of initial presentation. The breeds that were considered brachycephalic included boxer, bullmastiff, cane corso, Cavalier King Charles spaniel, Chihuahua, dogue de Bordeaux, English bulldog, French bulldog, Lhasa apso, Maltese, miniature pinscher, Pomeranian, pug, shih-tzu and Stafforshire bull terrier (1, 17–24). The brachycephalic dogs were further grouped into small (i.e., Cavalier King Charles spaniel, Chihuahua, French bulldog, Lhasa apso, Maltese, miniature pinscher, Pomeranian, pug and shih-tzu) and medium to large brachycephalic breeds (i.e., bullmastiff, cane corso, dogue de Bordeaux, English bulldog and Staffordshire bull terrier).

**Figure 1:**
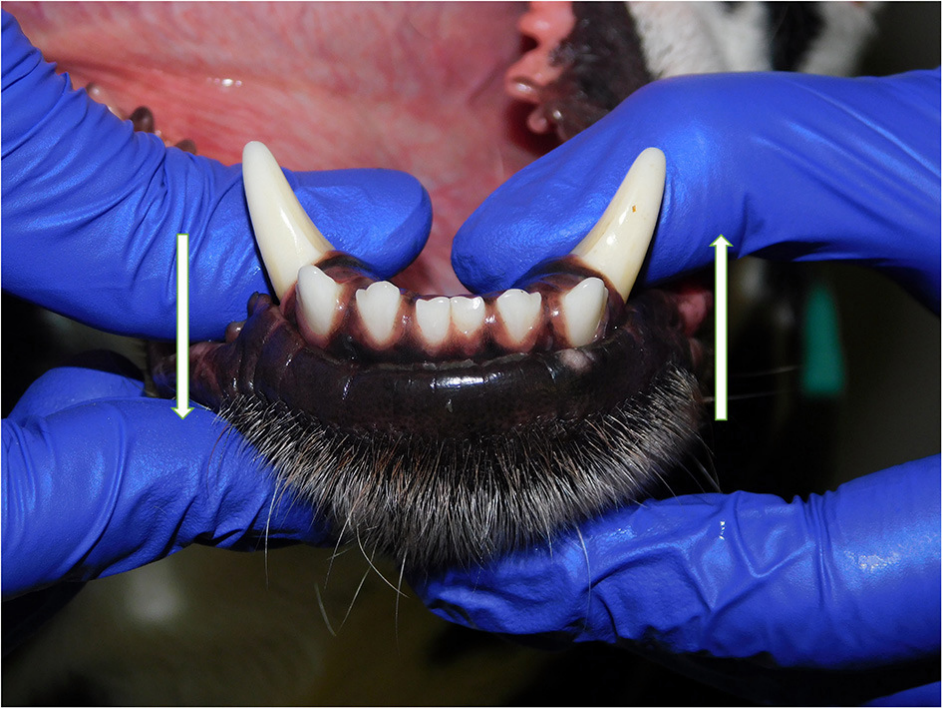
Dorsoventral mobility was evaluated by firmly holding right and left mandibles behind the canine teeth and pushing alternatively the mandibles in opposite directions (i.e., one mandible in ventral direction and the other one in dorsal direction, and vice versa).

**Figure 2:**
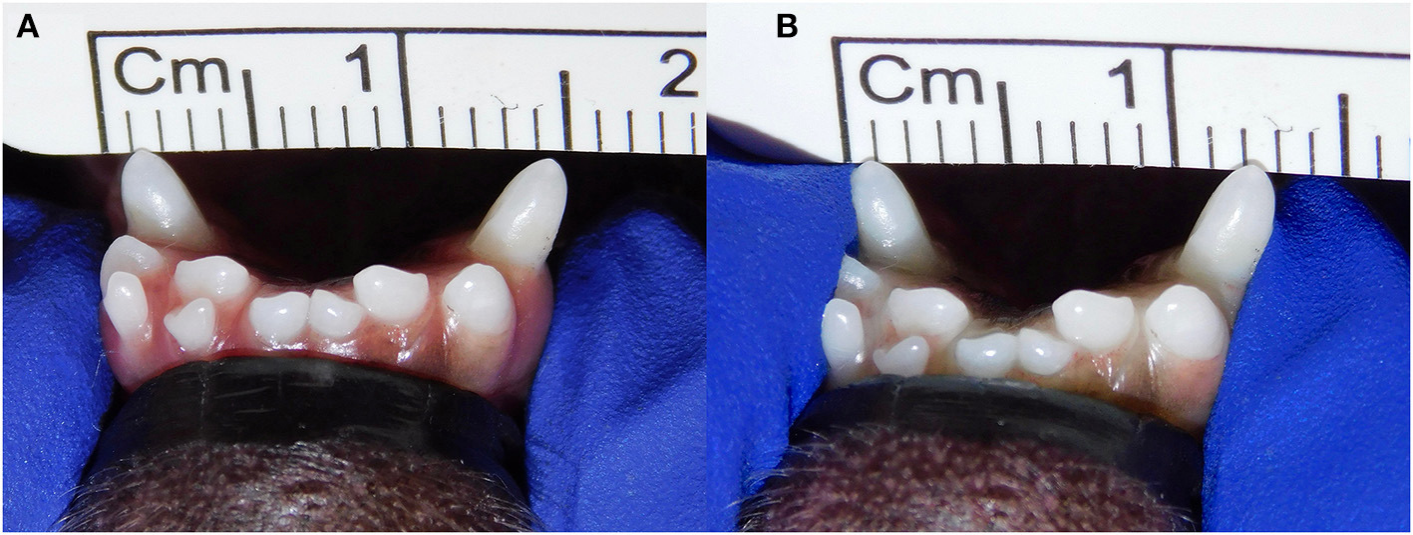
Example of lateromedial mobility evaluation in a clinical case. **(A)** Before applying any force; **(B)** approximation of the canine teeth by 2 mm (grade 2) after pressing the coronal tips with the thumb and index fingers of the same hand in a lingual direction. The index finger of the free hand is placed on the skin over the ventrocaudal aspect of the symphysis to better appreciate any slight movement.

**Figure 3:**
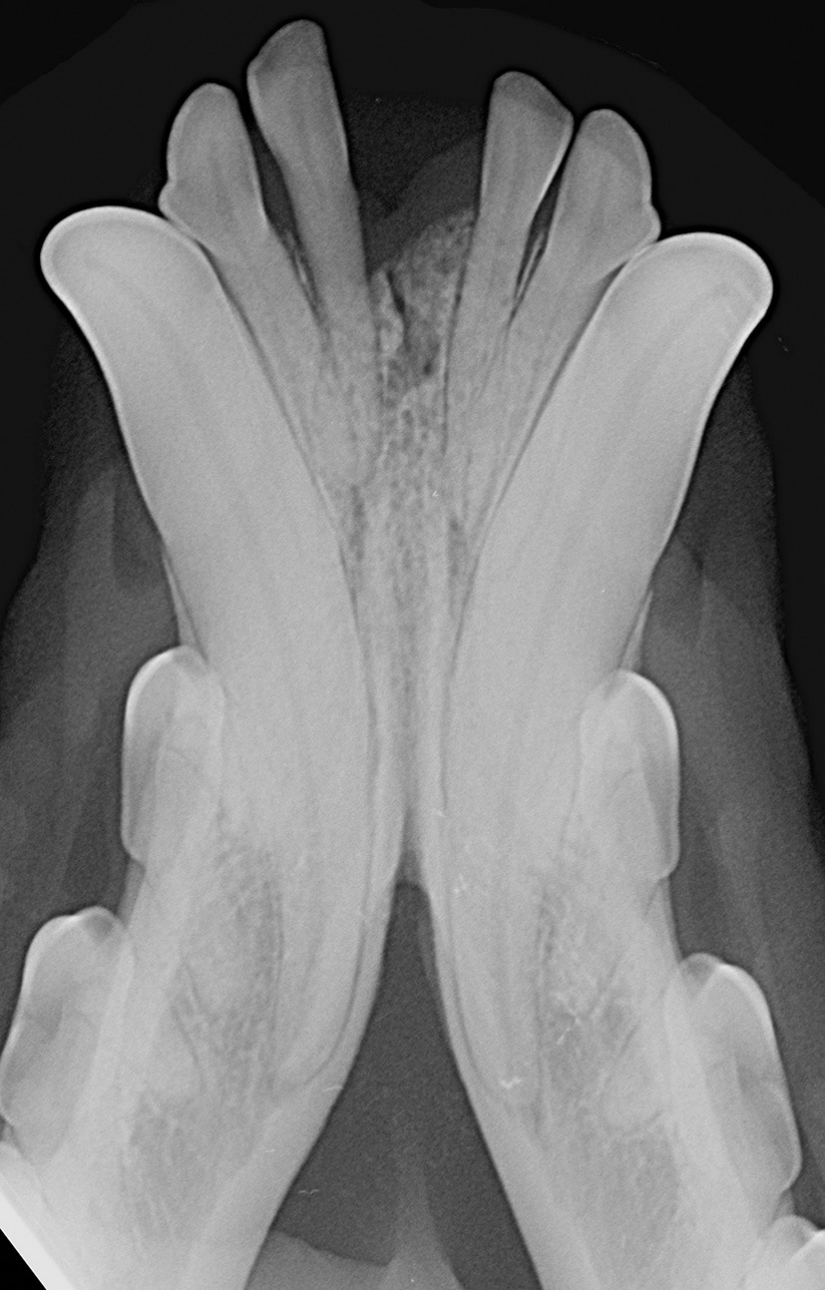
Radiopaque symphysis in 8 years-old, male castrated, Jack Russell terrier dog.

**Figure 4:**
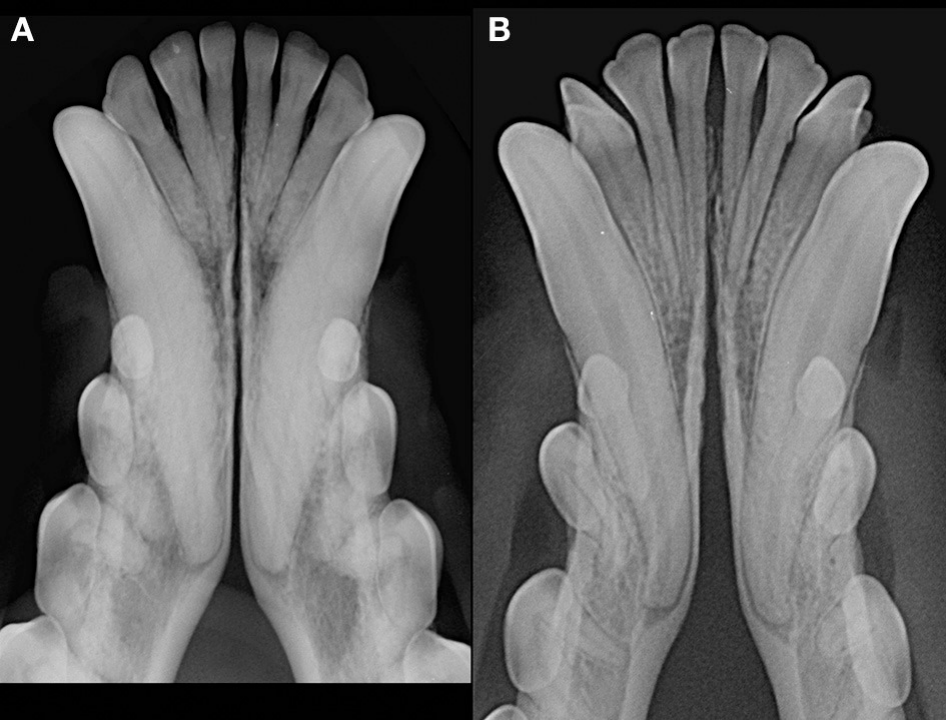
Radiographic appearance of open symphyses, with parallel [**(A)** 13 years-old, male intact, mixed breed dog] and divergent [**(B)** 19 months-old, male intact, toy poodle dog] margins.

Patients were excluded in case of recent or previous maxillofacial trauma involving the mandibles (cases with localized, mild maxillary trauma of known origin were included); neoplastic disease involving the mandibles rostral to the molar area, with the exception of benign lesions that did not cause consistent bone lysis or remodeling, such as small peripheral odontogenic fibromas; severe periodontal disease affecting canine teeth (i.e., AVDC stage 4: more than 50% of attachment loss) (25); absence of more than four mandibular incisor and/or one or both canine teeth; other diseases that caused severe bone lysis and remodeling (e.g., secondary hypoparathyroidism, or unerupted canine and/or incisor teeth with dentigerous cyst). Symphyseal mobility was evaluated in lateromedial (LM) and dorsoventral (DV) directions. During DV mobility evaluation, right and left mandibles were firmly held behind the canine teeth and alternatively pushed in opposite directions (i.e., one mandible in ventral direction and the other one in dorsal direction) (Figure 1). The degree of mobility was recorded as 0 (i.e., no mobility), 1 (i.e., independent movement of the mandibles with a ≤1mm variance at the level of the incisor teeth or the alveolar margin), 2 (i.e., independent movement of the mandibles with a 1≥3mm variance at the level of the incisor teeth or the alveolar margin) or 3 (i.e., independent movement of the mandibles with a >3 mm variance at the level of the incisor teeth or the alveolar margin). LM mobility was evaluated by pressing the coronal tip of right and left mandibular canine teeth with the thumb and index finger of the same hand in a lingual direction and visually evaluating any induced movement (i.e., approximation of the tip of the canine teeth) from the front (Figure 2). The placement of a finger of the free hand on the skin over the ventrocaudal aspect of the symphysis helped determining the presence of slight LM mobility. A grade 0 to 3 mobility scale was used (i.e., grade 0: no mobility; grade 1: ability to approximate the tip of the canine teeth by ≤1 mm; grade 2: ability to approximate the tip of the canine teeth 1 ≥3 mm; grade 3: ability to approximate the tip of the canine teeth >3 mm). If an animal was examined more than one time, time between visits and the different measurements were recorded and statistically evaluated. All clinical evaluations were performed by two operators (MG and SM). The radiographic examination of the mandibular incisive and symphyseal area was performed in all cases, but images were excluded from the statistical analysis if radiographic plate positioning, radiographic beam angle, and exposure were considered of insufficient quality to evaluate. The examination was performed on a single image obtained using the intraoral, rostrocaudal, bisecting angle technique for the mandibular canine teeth. Instrumentation included a dental radiographic machine (Gendex Oralix AC, Dental Systems, Milan, Italy) and CR (Computed Radiography) digital radiographic plates of variable sizes, based on patient's size (VistaScan, Dürr Dental SE, Bietigheim-Bissingen, Germany). All DICOM files were saved as JPG files and stored in a computer (MacBook Pro, Apple Inc., California, USA). Only brightness and contrast were digitally adjusted, if necessary. No other settings were modified. Radiographically, the symphysis was classified as open (i.e., symphyseal plates appearing separated by a radiolucent gap); or fused (i.e., the symphysis appearing partially or completely radiopaque) (Figure 3). Open symphyses were further described as having parallel (i.e., the margins are parallel to each other and to the midline, from the alveolar margin along the entire longitudinal symphyseal extension) (Figure 4A) or divergent margins (i.e., the margins are closer to each other at the alveolar margin and diverge progressively in a rostrocaudal direction) (Figure 4B). A blinded evaluation of the radiographic images was performed independently by four authors (i.e., SM, SB, MG, and EA) without information regarding the clinical grading. Cases classified differently by the evaluators were discussed and finally classified over a common consensus.

## 3 Statistical analysis

Data (i.e., age, sex, neutering status, breed, bodyweight, skull morphology as brachycephalic or non-brachycephalic, number of consults, degree of LM and DV symphyseal mobility, and radiographic appearance) was recorded in a commercially available spreadsheet, and statistical analyses were conducted using a commercially available statistics program (Stata version 14.2, Stata Corporation, College Station Texas, USA). Descriptive statistics were performed to report demographic data. Continuous data was assessed for normality by visualization of distributional plots and use of a Shapiro-Wilks normality test. When continuous data was normally distributed, means and standard deviations were reported; otherwise, medians and overall range were reported. Totals and percentages were used to describe categorical data. Associations between categorical data were evaluated using a Fisher's exact test. To explore factors that affected mobility in LM or DV directions and the radiologic score, ordinal logistic regression was evaluated. *P* < 0.05 were considered significant.

## 4 Results

Five hundred and sixty-seven dogs of 95 different breeds were included in the study, accounting for a total of 695 clinical evaluations (Supplementary Table 1). None of the continuous variables were normally distributed. The most common breeds (i.e., ≥10 dogs) were mixed breed (126 dogs, 22.2%), Jack Russell terrier (31 dogs, 5.5%), dachshund (29 dogs, 5.1%), cocker spaniel (27 dogs, 4.8%), boxer (24 dogs, 4.2%), toy poodle (23 dogs, 4.1%), Maltese (19 dogs, 3.3%), Chihuahua (17 dogs, 3.0%), Labrador retriever (16 dogs, 2.8%), golden retriever, German shepherd, Border collie (14 dogs each, 2.5%), and American Staffordshire terrier (10 dogs, 1.8%). Two hundred and thirty six dogs (41.6%) were intact males, 84 (14.8%) neutered males, 78 (13.8%) intact females and 169 (29.8%) neutered females. Bodyweight was available for 564 cases and ranged from 0.8 kg to 79 kg, with a median of 14.4 kg. Age at presentation ranged from 3 months to 16 years and 4 months, with a median of 6 years and 9 months. 439 dogs (77.4%) were evaluated only once, 100 dogs (17.6%) twice, 17 dogs (3.0%) three times, 7 dogs (1.2%) four times, 2 dogs (0.4%) five times, 1 dog (0.2%) six times and 1 dog (0.2%) seven times. The median period of time between different evaluations in these cases was 7 months, with a range from 2 weeks to 6 years and 1 month. The elapsed time between evaluation was not standardized.

**Figure 5:**
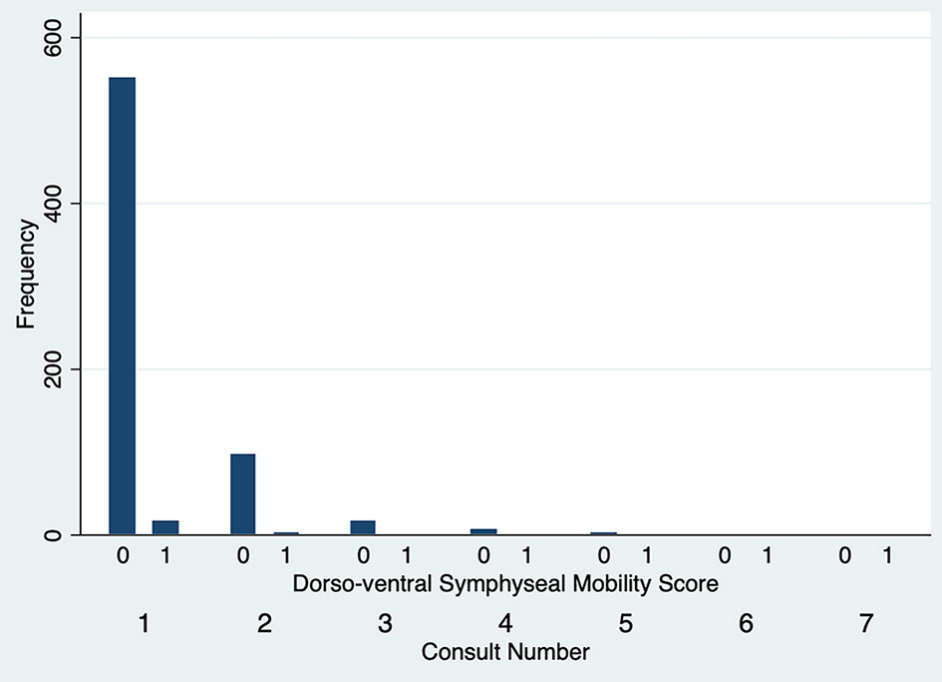
Dorsoventral symphyseal mobility score frequency over subsequent evaluations.

**Figure 6:**
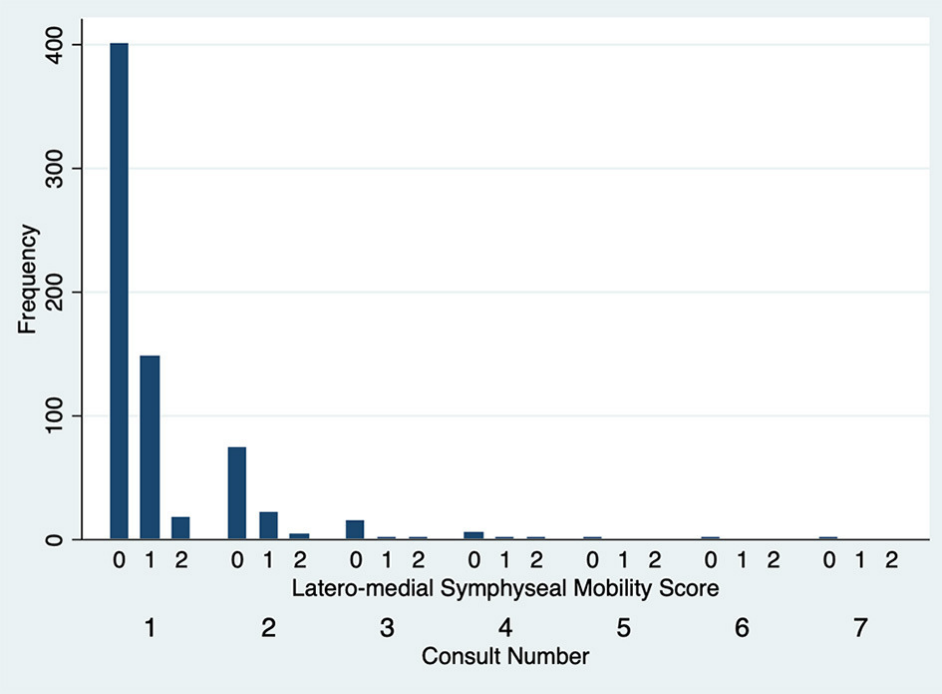
Lateromedial symphyseal mobility score frequency over subsequent evaluations.

The degree of DV symphyseal mobility at the first evaluation was determined to be 0 in 551 cases (97.2%) and 1 in 16 cases (2.8%). For cases evaluated twice, at the second examination DV mobility was 0 in 97 cases (97%) and 1 in 3 cases (3%). DV symphyseal mobility did not vary statistically between the first two evaluations (Fisher's exact test *P* = 1.00) (Figure 5) with only two cases increasing from a DV mobility score 0 to 1 and two cases decreasing their mobility score from 1 to 0. None of the dogs ever showed a DV mobility score of 2 or 3. The degree of LM symphyseal mobility at the first evaluation was determined to be 0 in 401 cases (70.7%), 1 in 148 cases (26.1%) and 2 in 18 cases (3.2%). For cases evaluated twice, at the second examination it was 0 in 74 cases (74%), 1 in 22 cases (22%) and 2 in 4 cases (4%). LM symphyseal mobility did not vary statistically between the first two evaluations (Fisher's exact test *P* = 0.42) (Figure 6) with eight cases showing increased LM mobility between evaluations and 6 cases showing decreased LM mobility between their first and second evaluations. None of the cases ever showed LM mobility 3. There was not a significant change in mobility score over time for the cases that were examined more than once (Fisher's exact test *P* = 0.71).

83.8% of the radiographs were considered of acceptable quality for evaluation. Of these 475 images, two cases (0.4%) were classified as having a fused symphysis and 473 (99.6%) an open symphysis. The margins of the open symphyses were considered divergent in 355 cases (74.7%) and parallel in 118 cases (24.8%). When investigating radiographic changes over time for cases that were examined more than once, there were no significant differences found (*P* = 0.33).

An ordinal logistic regression model exploring factors that affected DV symphyseal mobility was statistically significant (*P* < 0.0001), with an increase in DV mobility being associated with an increase in LM mobility (*P* < 0.001, OR 9.72, 95% CI 3.48–27.11). An ordinal logistic regression model exploring factors affecting LM symphyseal mobility was also statistically significant (*P* < 0.0001), with an increase in LM mobility being associated with an increased DV mobility (*P* < 0.001, OR 11.38, 95% CI 3.75–34.56). With an increase in age there was an overall statistical decrease in symphyseal LM mobility (*P* < 0.001, OR 0.99, 95% CI 0.99–0.99). An increase in bodyweight was also significantly associated to a decrease in LM mobility (*P* < 0.001, OR 0.92, 95% CI 0.90–0.94). Furthermore, there was a greater chance in the young dog group (75 cases) than the mature dog group (491 cases) of having any mobility (*P* < 0.001, OR 0.29, 95% CI 0.18–0.47), DV (*P* = 0.002, OR 0.20, 95% CI 0.07–0.54) or LM (*P* < 0.001, OR 0.31, 95% CI 0.19–0.50) mobility. Radiographically, the symphysis appeared more frequently divergent in young dogs and parallel in mature dogs (*P* = 0.001, OR 7.86, 95% CI 2.42–25.47). With an increase in bodyweight a decrease in mobility in both directions was shown (*P* < 0.001, OR 0.92, 95% CI 0.90–0.94). The model was statistically significant when investigating separately bodyweight subgroups for any mobility (*P* < 0.0001, OR 0.40, 95% CI 0.33–0.49), with 30.2% of dogs in subgroup 1, 69.7% of dogs in subgroup 2, 79.6% of subgroup 3 dogs and 87.3% of subgroup 4 dogs showing no mobility. The same was shown when evaluating DV and LM mobility separately, with no DV mobility in 92.5%, 96.1%, 99.5%, and 98.6% of cases (*P* = 0.006, OR 0.49, 95% CI 0.29–0.81) and no LM mobility in 29.2%, 70.2%, 79.6% and 87.7% of cases (*P* < 0.001, OR 0.39, 95% CI 0.32–0.48) in bodyweight subgroups 1 to 4, respectively. The radiographic appearance changed significantly from divergent to parallel as bodyweight increased (*P* = 0.003, OR 1.38, 95% CI 1.12–1.70). This finding was also confirmed when analyzing young dogs alone (*P* = 0.005, OR 1.96, 95% CI 1.23–3.12) but not when investigating adult dogs alone (*P* = 0.16). The logistic regression model associating age and bodyweight showed a significant statistical reduction in young dogs (< 15 months of age) for any mobility (*P* = 0.02, OR 0.61, 95% CI 0.40–0.92) and LM mobility (*P* = 0.005, OR 0.54, 95% CI 0.35–0.83), but not for DV mobility (*P* = 0.12), with an increase in bodyweight. The same model in mature dogs (>15 months of age) showed a statistically significant decrease in any mobility (*P* < 0.0001, OR 0.37, 95% CI 0.29–0.47) and LM mobility (*P* < 0.001, OR 0.37, 95% CI 0.29–0.47), but not in DV mobility (*P* = 0.09), with an increase in bodyweight.

Brachycephalic dogs accounted for 18% (102) of the cases. The degree of DV mobility at the time of the first evaluation was 0 in 94 cases (92.2%) and 1 in 8 cases (7.8%), and the degree of LM mobility was 0 in 62 cases (60.8%), 1 in 35 cases (34.3%) and 2 in 5 cases (4.9%). Being a brachycephalic dog was associated with having an increase in DV mobility score compared to the rest of the population (*P* = 0.01, OR 4.84, 95% CI 1.44–16.18). Being a brachycephalic dog was not associated with having an increase in the LM mobility score (*P* = 0.87). Being a small size brachycephalic breed was associated with higher mobility in any direction (*P* < 0.001, OR 0.07, 95% CI 0.02–0.24) and in LM direction (*P* < 0.0001, OR 0.04, 95% CI 0.01–0.20), but not in DV direction (*P* = 0.19), as compared to larger brachycephalic breeds. Radiographs were of sufficient quality to evaluate in 90 of the 102 brachycephalic dogs (88.2%). Seventy nine (87.8%) were classified as divergent, 11 (12.2%) were classified as parallel and none as fused. The divergent type was significantly more common in brachycephalic than in non-brachycephalic dogs (*P* = 0.003). No association between clinical mobility and radiographic class was shown in brachycephalic dogs as a group (small and medium/large size) (*P* = 0.23).

No variation in clinical mobility or radiographic appearance was shown for any single breed or sex/neutering status in the ordered logistic regression model. Differences between dogs of each of the most represented breeds and the whole population were also not statistically significant in this model.

## 5 Discussion

### 5.1 Anatomy, physiology and classification

Anatomically, the canine mandibular symphysis is composed by right and left mandibular plates (e.g., the articulating surfaces of the rostral area of the mandibular bones), separated by the symphyseal space (2). The dorsal margin is in continuation with the symphyseal surface of the mandible (or symphyseal shelf), that is bounded by the incisor, canine and first/second premolar teeth. The mandibular plates have the shape of an arrow, pointing rostrally. The dorsal border extends from the alveolar margin mesial to the first incisor tooth to a variably defined tubercle present in an area of projection between the first and second premolar teeth. The ventral border shapes the rostral profile of the mandible, running caudally to a more developed tubercle ventral or slightly ventrocaudal to the dorsal one. Finally, the caudal margin is indented by a wedge-shaped notch, where the genioglossus, mylohyoideus and geniohyoideus muscles attach (2, 26). The symphysis on the dorsal aspect is covered by mucoperiosteum, intimately associated to a tight fibrous joint capsule. On the labial side, between the first incisor teeth, it is covered by the gingival tissue and alveolar mucosa and submucosa. The ventral aspect is also further covered by muscular attachments as well as subcutaneous and cutaneous tissues. Within the symphyseal space a single fibrocartilage pad, abundant fibrous connective tissue, a number of ligaments, and a venous plexus are present. The arterial supply is composed by the mental and incisal branches of the inferior alveolar artery rostrally and the submental artery caudally (2). The innervation is composed by the lingual, the inferior alveolar, the incisal, the mental and the mylohyoid nerves (1–3). The central part of each symphyseal plate may show bony ridges and valleys, with a distribution that is opposite to the other side (2). The fibrocartilagineous pad is located dorsorostrally, at the level of a smooth area of the symphyseal plates, and has a cuneiform shape, being wider dorsally than ventrally on transverse section, and wider rostrally than caudally in coronal section. Its function is to keep the symphyseal plates apart, and to resist mandibular rotation. Some of the fibrous tissues within the symphyseal space form the superior transverse, inferior transverse, inferior oblique and internal cruciate ligaments. The internal cruciate ligaments attach in a central triangular area of the symphyseal plates, and have been described to strongly resist dorsoventral and rostrocaudal plate displacement, allowing only some rotational movements (2). On the other hand, the dorsal joint capsule, together with the superior and inferior transverse and inferior oblique ligaments, resist the lateral separation of the symphyseal plates (2). Based on an observational and microscopic study on skeletal material from a museum collection and cadaveric specimens, the carnivores' mandibular symphysis was separated into in four classes (3). Class I symphyses were characterized by flat symphyseal plates, possibly only showing a few, low irregularities. The symphyseal space was wider caudally than rostrally and the soft tissues were structured as described above. Class II symphyses showed plates separated by a narrow space that had approximately the same width all along the symphysis, and ridges and valleys were more numerous and intimately related than in class I. A thin fibrocartilaginous pad, and thick and short fibrocartilaginous and connective fibers running nearly transversely across the joint, were described. The plate irregularities in class III symphyses were taller and interdigitated more that in class II, and a smooth area was small or absent. The fibrocartilaginous pad was smaller, the ligaments fibers were mostly transverse and caudally irradiated in all directions. Finally, bony fusion of the symphyseal plates characterized class IV symphyses, with non-lamellar bone obliterating the joint space. These classes also correlated with a symphyseal mobility score that included a “maximum flexibility,” with basic movements between the mandibles at the symphysis visible to the naked eye and manually easy to produce (typical of class I symphyses); a “limited flexibility,” with joint movements visible, but manually more difficult to produce than in maximally flexible joints (typical of class II symphyses); a “stiff” symphysis, that allowed only minute amounts of visible movements under forceful manipulation (typical of class III symphyses); and a “rigid” symphysis that allowed no visible movements (typical of class IV symphyses) (3). Lateral and medial mobility were evaluated on one side at a time by turning the tip of each canine tooth in both directions, and rostrocaudal and dorsoventral mobility by attempting to slide one symphyseal plate over the other. Therefore, the described technique has similarities but is not identical to our technique. In particular, we did not evaluate the rostrocaudal or the medio-lateral symphyseal mobility. In the present study we attempted to find a more objective and clinically applicable way to evaluate symphyseal mobility, even though we recognize that the proposed scoring system still holds a significant level of subjectivity, both in the force applied to the mandibles during examination as well as in the actual measurement in millimeters. Based on the studies by Scapino, dogs have been described as having a class I symphysis, with no mobility in cranio-caudal and dorsoventral directions but a high degree of flexibility in lateral and medial directions (3). In fact, the symphysis in this species has been described as the “third mandibular joint,” given that a flexible symphysis may synergistically collaborate with the temporomandibular joints during function, e.g., allowing some medio-lateral rotation of the mandible on the active side during mastication, to better align carnassial teeth for shearing and molar teeth for crushing (2, 3). The results of our study partially differ from Scapino's results, as the great majority of our cases did not show any clinical mobility in LM direction, and few cases (2.8%) showed some DV mobility. These differences could be explained by a greater anatomical variation than has been described in the past, and/or a greater symphyseal mobility in live animals as compared to anatomical specimens due to a different degree of hydration of the anatomical structures. It should also be considered that both Scapino's and the proposed mobility scales hold some degree of subjectivity in mobility perception and scoring. Also, we showed that in dogs there is slightly greater mobility in LM rather than DV direction, possibly indicating the presence of tighter dorsal ligaments and dorsal joint capsule as compared to ventral structures. In carnivores with a stiffer symphysis such as the large members of the Felidae and Ursidae, the fibrocartilage pad is narrower, and the symphyseal ligaments are shorter and differently oriented (3). This type of stiffer symphysis may be an adaptation to the need for increased masticatory forces required to fragment prey eaten by these animals, and may also make possible concomitant bilateral use of the jaw musculature, generating higher forces at the working side (3). More anatomical and histological studies are warranted in dogs to better describe the ligamentary structure and explain the differences between individuals that show different degrees of symphyseal mobility.

### 5.2 Radiographic evaluation

In this study radiography was the imaging modality of choice, as it still represents the most commonly used imaging modality for dental patients. It is important to highlight, though, that the two dimensional depiction of the symphysis only allows limited information to be collected and that even mild changes in x-ray beam angulation could affect the radiographic appearance of the area (15). A number of images were excluded because they were considered of insufficient quality, due to asymmetry in latero-lateral direction and/or due to an excessively obtuse angle to the radiographic plate (i.e., the x-ray beam aimed more perpendicularly to the plate rather than to the bisecting angle useful for canine teeth evaluation, with consequent foreshortening of the obtained image). The use of advanced modalities such as computed tomography and cone beam computed tomography could have certainly added further insights into the evaluation of the shape, size and roughness of the symphysis, while avoiding most of the radiographic limitations.

**Figure 7:**
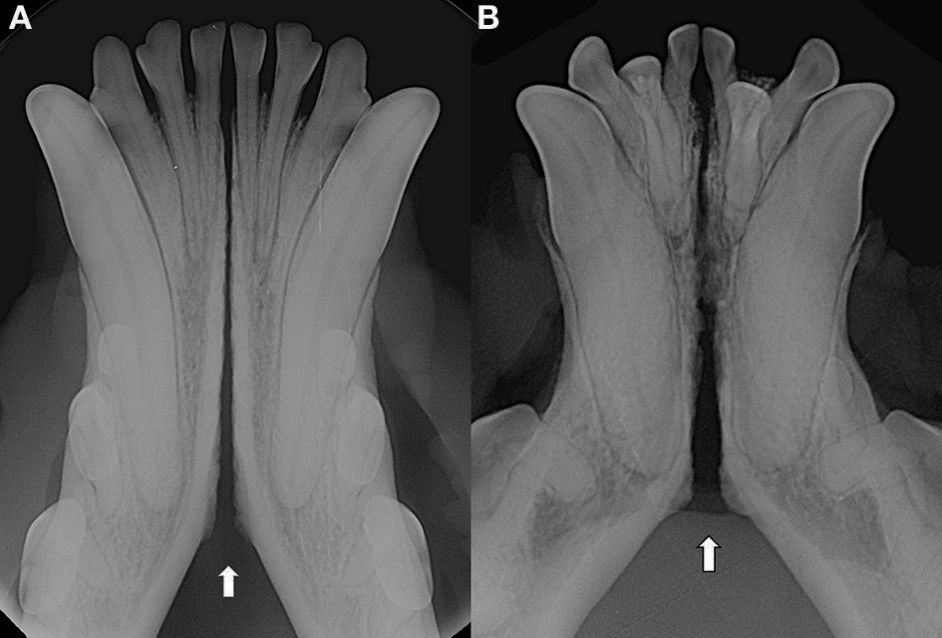
Examples of cases with a relatively clear change in angulation of the mandibular ventral cortex, identified as the caudal extension of the symphysis, in a 3 years-old, male intact, Australian cattle dog (mesocephalic) **(A)** and a 5 years-old, female neutered, French bulldog (brachycephalic) **(B)**.

**Figure 8:**
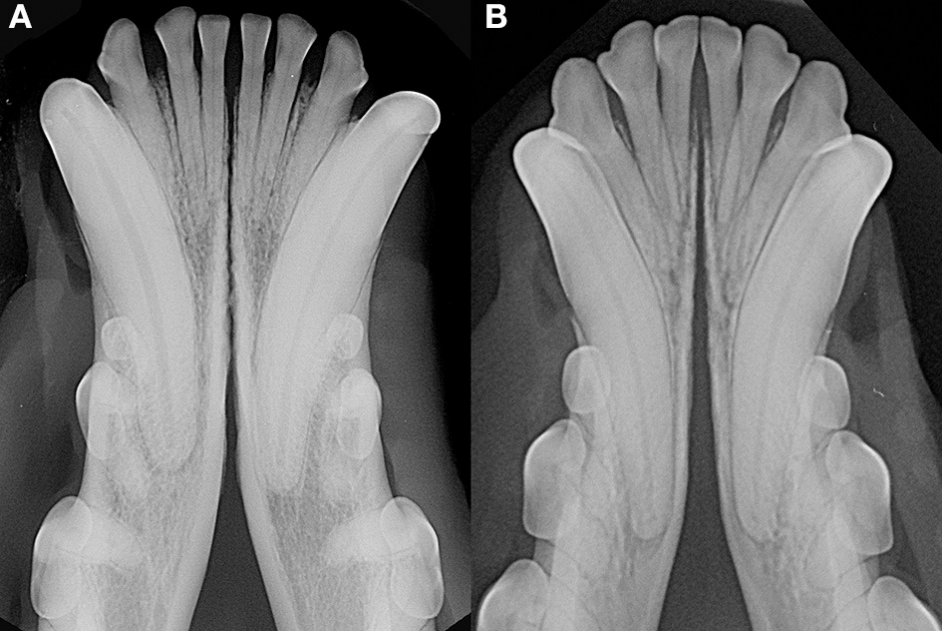
Examples of cases with a smooth transition from the symphyseal area to the ventral cortex of the mandibular body in a 6 years-old, male castrated, mixed breed dog **(A)** and in a 5 years-old, female neutered, dachshund dog **(B)**.

As previously observed (3), often the symphyseal space appears divergent, i.e., wider caudally than rostrally. However, almost 25% of our cases had radiographically parallel symphyseal plates, more similarly to Scapino's description of class II symphyses (3). It is possible that we found more variation because we evaluated a larger population as compared to the number of dogs studied by Scapino (2, 3). Also, we should consider that breed selection, known to have modified skull shape of certain breeds significantly over the years (20, 27, 28), could have also modified the average symphyseal shape. We acknowledge that it would have been interesting to also classify the radiographs based on other factors, such as symphyseal plate irregularities and width of the symphyseal space, and correlate these data with clinical mobility. The study authors initially attempted to translate Scapino's anatomical classification system into a similar radiographic classification system (unpublished data). However, reaching a common consensus between evaluators was not possible for many radiographic images, particularly when trying to differentiate between the degrees of irregularities present. Furthermore, as images were saved in JPG format rather than as DICOM files we were unable to take exact measurements of the radiographic width of the symphysis, and therefore to have an objective method to discriminate between thin and wide symphyseal gaps. In an attempt to restrict the inherent subjectivity of the radiographic evaluation, we proposed a simpler two-factor (i.e., open and fused) classification. However, even the description of the symphyseal shape (i.e., parallel or divergent) was not always straightforward. In fact, in some images the exact caudal extension of the mandibular symphysis was unclear. In certain cases, in an area that varies from the area of projection of the canine tooth apex to the area of projection of the third premolar distal root apex, there is a relatively clear angle of the mandibular ventral cortex, that we identified as the possible caudal extension of the symphysis (Figure 7A). This anatomical landmark was more evident in some brachycephalic dogs (Figure 7B). However, in other cases the symphyseal plates appeared to smoothly transition into the ventral cortex of the mandibular body, without an obvious radiographic difference in shape or angulation (Figure 8), which made determination of the caudal extent of the mandibular symphysis difficult. Further anatomical and histological studies defining the exact extent of the mandibular symphysis would improve our understanding of the possible variations of this anatomical area.

### 5.3 Age

**Figure 9:**
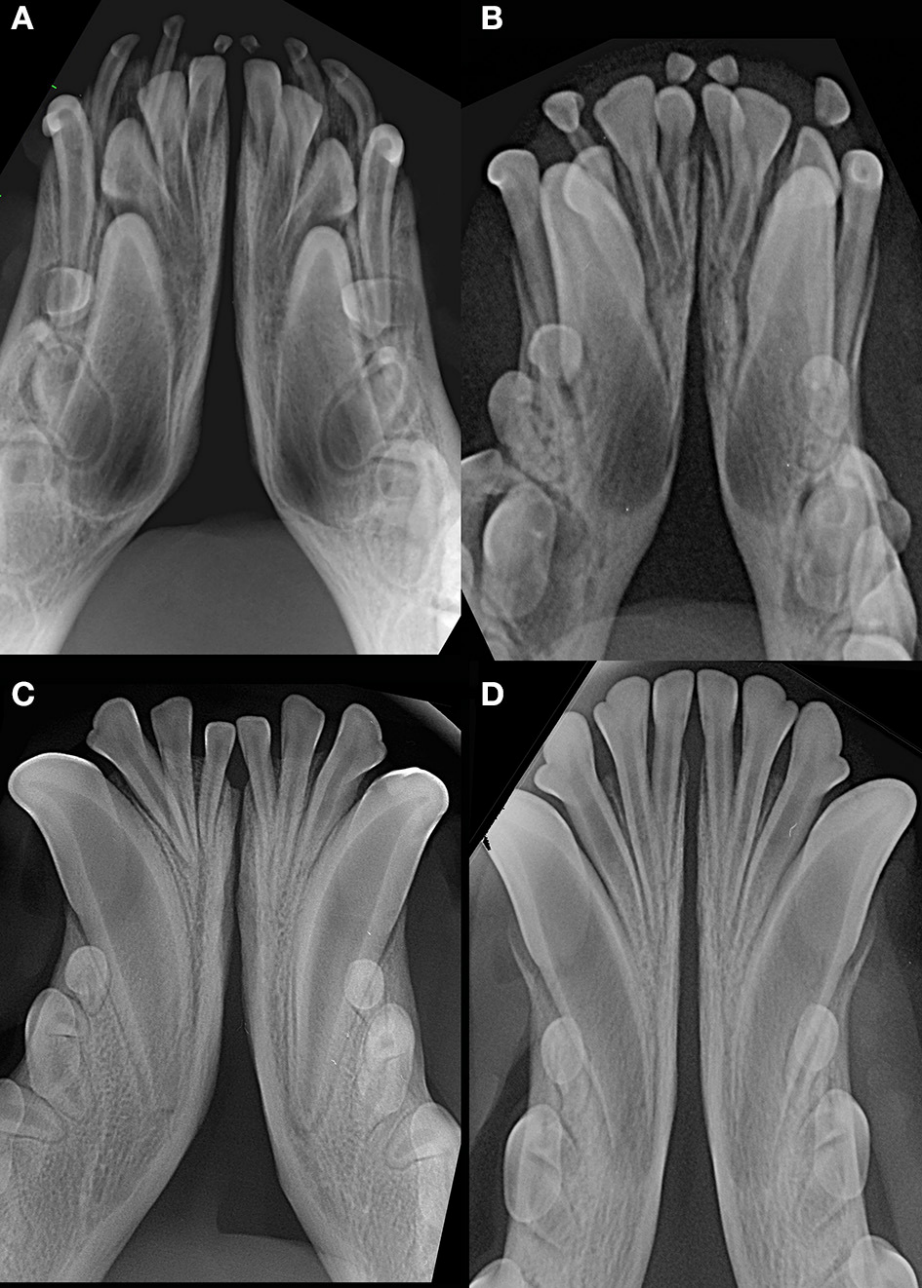
Examples of young dogs with subjectively wide open symphysis. **(A)** 3 months-old, male intact, American Staffordshire terrier; **(B)** 5 months-old, female intact, Maltese; **(C)** 7 months-old, male intact, English Staffordshire bull terrier; **(D)** 8 months-old, female intact, mixed breed.

The symphysis of large, young, healthy dogs has been described as a “narrow firm union with virtually no mobility” (15). Another source describes it in young patients as a thick radiolucent line (16). In the present study, the symphysis of young animals appeared with an obvious, often divergent radiolucent gap interposed between the symphyseal plates (Figure 9), and clinically with a higher mobility score than in the rest of the study population. However, the mobility decreased with an increase in bodyweight, in the whole population as well as in young and mature dogs when evaluated separately as subgroups. It is notable, on the other hand, that DV mobility did not differ significantly in the age subgroups, indicating that DV mobility was more consistent (i.e., rarely present) throughout the study population. Studies performed on mice focused on changes of symphyseal mineralization during their lifetime, with evidence of unfused mandibular symphysis in young animals compared to more fused symphysis in adult and old animals (11–13). The main hypothesis for these changes (i.e, development of bony irregularities on the symphyseal plates, of thicker cortical bone and greater bone density) involve skeletal and soft-tissue responses induced by dietary changes in the post-weaning period (12, 13). Also, an increased masticatory muscle activity may cause load-induced degradation of the fibrocartilage pad and connective tissue, and lead to a compensatory osteogenic production in adult rabbits (12, 13). The same concept has been reported in dogs (15). However, it has to be considered that masticatory forces and chewing habits of domesticated dogs differs quite drastically from those of wild carnivores and especially from herbivores.

**Figure 10:**
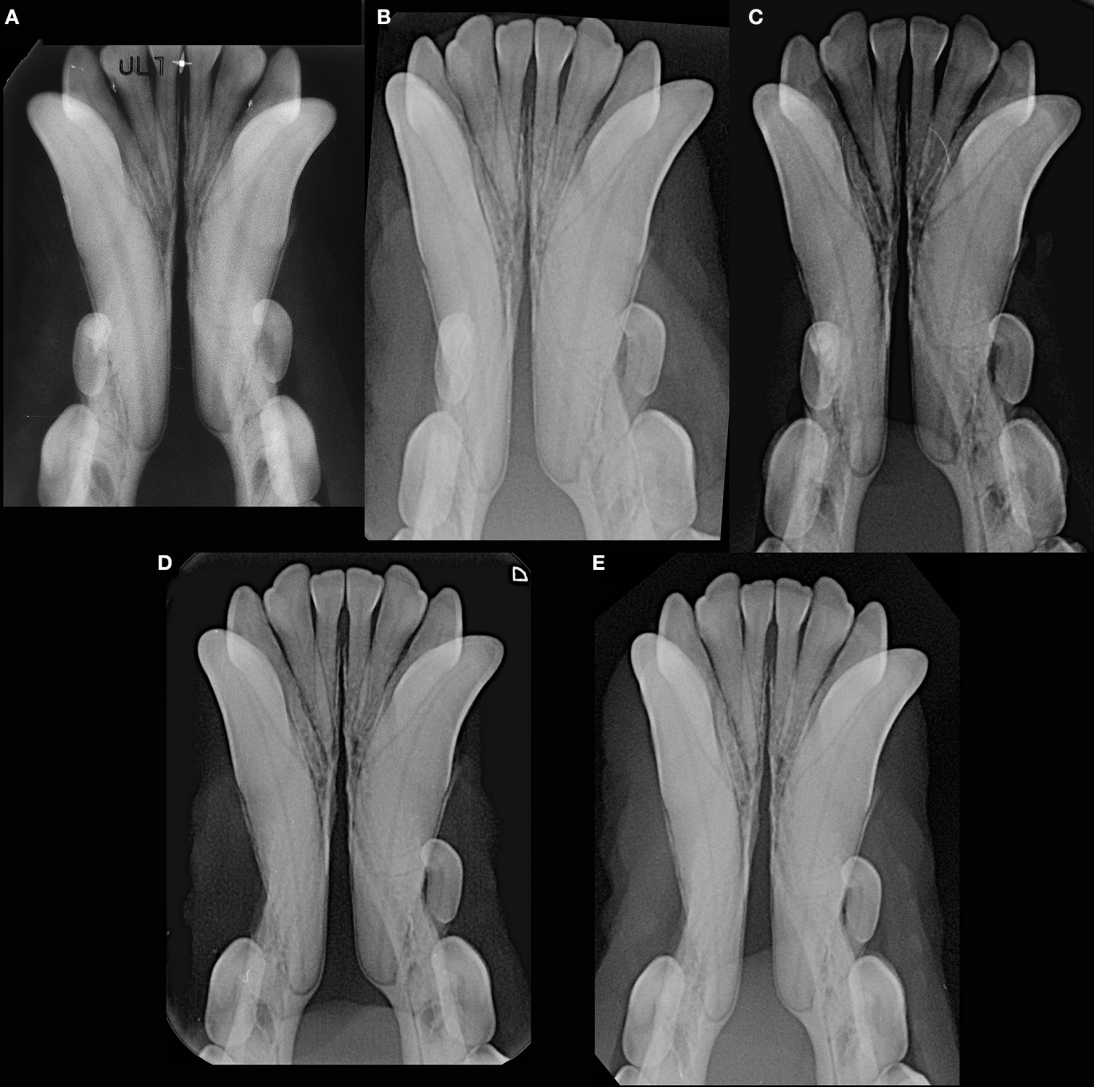
Example of a male intact, toy poodle dog evaluated at 1 year and 7 months (before the start date of the study) **(A)**, 2 years and 9 months **(B)**, 5 years and 9 months **(C)**, 6 years and 3 months **(D)**, and 7 years and 1 month **(E)** of age, showing lack of changes in radiopacity and symphyseal width over time. This dog showed clinical mobility DV = 0, LM = 2.

In our study population, no statistical difference in clinical mobility or radiographic appearance was found over time for any single case, indicating that in normal conditions ligamentary stiffness and thickness are unlikely to change over time (Figure 10). It is also possible that we have been unable to show such changes because only about a quarter of the patients included in this study were evaluated more than once, and the elapsed time between evaluation was not standardized.

Interestingly, we found a fused symphysis in 8 years-old, male castrated, Jack Russell terrier dog (Figure 1) and in a 13 years-old, intact male, dachshund dog. They were presented and treated for mild ulcerative stomatitis affecting the mucosa of the cheeks, and oronasal fistulas of periodontal origin affecting the right maxillary canine tooth, respectively. Previous trauma or other diseases that could have affected the mandibular symphysis were not reported by the owners, but cannot be totally excluded. Also, these cases were evaluated only once in their lifetime, therefore it was not possible to evaluate any changes in time. Still, even if rare, we should consider radiographically fully ossified symphyses as possible in dogs.

### 5.4 Bodyweight and size

No statistical difference in mobility score was found for any specific breed in the present study, but our data suggest that an increase in bodyweight relates to an increase in symphyseal stiffness. This may be due to a different arrangement of the symphyseal ligaments, a tighter joint capsule, and a thinner symphyseal pad as compared to small and medium size dogs. In fact, bite force has been shown to increase as cranial size increases (29, 30), which may translate to a need for a stiffer symphysis. However, as the body condition score of the patients was not evaluated, differences in bodyweight may not directly correlate to increased skeletal size. We also evaluated potential differences between small breeds, independently from their skull morphology, and the rest of the study population, as the symphysis of small dogs has been described as being wider, and with varying degrees of mobility (15, 16). To be able to include also mixed breed dogs in the statistical analysis, the whole population was divided into bodyweight subgroups. It was confirmed that an increase in bodyweight was associated with a decrease in mobility and by a more frequent parallel radiographic appearance. Conversely, smaller dogs showed a clinically more mobile and radiographically more radiolucent and divergent symphysis. However, it should also be noted that the two cases with a radiographically fused symphysis were both of small breeds (i.e., a Jack Russell terrier and a dachshund).

### 5.5 Breeds and skull morphology

Variation in skull shape among different dog breeds have been associated with differences in jaw strength and biting forces (29, 30). Bite force increases from the dolichocephalic to the brachycephalic morphology, possibly due to the fact that the out-lever arm of the mandible in dolichocephalic skulls is longer than in brachycephalic individuals (29–31). Some theories support the fact that animals with higher biting forces may need a stiffer symphysis, and symphyseal fusion obviously stiffens the symphysis (3, 9, 10). A fused symphysis would allow the transfer of higher occlusal forces from the balancing to the working side of the mandibular bodies during mastication (3, 9, 10). So, in an attempt to evaluate possible differences, we compared the brachycephalic dogs (18% of the cases) to the rest of the study population. Interestingly, brachycephalic dogs showed a divergent symphysis more commonly than non-brachycephalic dogs. Furthermore, brachycephalic breeds as a group showed a higher mobility in DV direction as compared to the rest of the population. These findings are in contrast with the expectation of a stiffer symphysis in these dogs. It is important to note, though, that in small dog breeds the shape of the skull does not seem to be a significant factor in determining bite force (29), and most of our brachycephalic patients were actually of small size. When small brachycephalic breeds and larger brachycephalic breeds were analyzed separately, there was no statistical difference in DV mobility between the two groups, but in smaller brachycephalic dogs a higher symphyseal mobility in LM direction was shown, confirming once more that DV mobility is generally less variable, possibly due to a tight dorsal ligamentous component. The altered shape of the mandible often present in brachycephalic dogs, with dorsal and/or lateral bowing of the mandibular bodies, may influence the symphyseal shape too, which may result in the divergence of the symphyseal plates which we observed more frequently in the brachycephalic dogs in this study.

We acknowledge the fact that breed classification based on skull morphology (i.e., dolicocephalic, mesocephalic and brachycephalic) may vary based on different studies and type of indices used, with some breeds being classified differently over time (1, 20, 27). Also, there is individual variation within a breed, which could not be accounted for in the present study. Furthermore, we elected to exclude mixed breed dogs from this statistical analysis, as we were unable to retrieve exact information on the skull morphology of these patients. Therefore, our results related to brachycephalic dogs should be confirmed in future studies, with more precise skull measurements and classification of patients.

### 5.6 Clinical application

A better understanding of normal variations in mandibular symphyseal anatomy can be beneficial to clinicians for treatment planning purposes. As shown in the present study, even in healthy canine patients some symphyseal mobility may be expected, and does not implicate the necessity for treatment. The complex architecture of the symphyseal fibers allows compensation and a certain resistance to shear (i.e. dorsoventral and rostrocaudal forces), bending and compression stresses that occur during normal prehension and mastication (2–5). Importantly, none of the included dogs showed DV mobility score higher than 1, or LM mobility score higher than 2. Therefore, a higher score may indicate the presence of a pathological process and the need for intervention.

In particular, a major force resulting from trauma can lead to a partial or more usually complete tear of symphyseal ligaments and mucoperiosteum, resulting in separation and increased mobility at the symphysis. In fact, the mandibular symphyseal area is often involved in craniomaxillofacial traumatic events, frequently in association with other concomitant maxillofacial fractures (15, 32, 33). In dogs, symphyseal separation has been reported in 33.9% of cranio-maxillofacial trauma cases (34, 35). After separation or fracture, stabilization of the site for about 6 weeks is recommended, and different techniques have been advocated in order to restore function and occlusion (32, 33). The presence of neoplasia, advanced periodontitis and metabolic diseases may cause pathologic jaw fractures (15, 33), and may alter the mobility and radiographic appearance of the symphysis (15), but new studies are necessary to investigate the effect of these conditions on the mobility and anatomy of the symphysis. Also, to date it is unknown if changes to and eventually fusion of the symphysis may occur during the healing process following any of these diseases.

Dysplasia of the temporomandibular joint (TMJ) is considered to predispose some animals to open mouth jaw locking (36–43). A certain flexibility of the symphysis may also play a role in the development of this condition, allowing slight lateral rotation of the involved mandible. However, during the study period only one dog was presented for open mouth jaw locking. This patient, a basset hound with tomographic signs of TMJ dysplasia, did not show symphyseal mobility in either direction. Therefore, we cannot make any conclusion on the role of symphyseal morphology in this condition.

## 6 Conclusions

The main aim of the study was to describe and classify the normal range of variation in clinical mobility of the mandibular symphysis in dogs using a standardized classification system and radiographic evaluation. Further, the study examined factors that could affect these scores. It was shown that the majority of cases had little to no mobility, but a certain degree of clinical mobility can be expected in some cases, and that when mobility in one direction is present, some mobility in the other direction is also likely. However, DV mobility score higher than 1 and a LM mobility score higher than 2 may indicate the presence of disease. It is also expected that a stiffer symphysis may be found in older individuals, particularly in large breed dogs.

Radiographically, the mandibular symphysis appears radiolucent in the majority of dogs, but bony fusion was rarely seen. The shape is very commonly divergent in a rostrocaudal direction, but particularly in large breed dogs the symphyseal plates may also appear parallel. Compared to the rest of the population, brachycephalic dogs showed a slightly higher mobility in DV direction and a more commonly divergent symphysis.

To better characterize the symphyseal morphology, improve the proposed classification and describe possible variations in case of pathology, further studies are warranted, possibly using advanced imaging modalities and histological evaluations.

## Data availability statement

The original contributions presented in the study are included in the article/Supplementary material, further inquiries can be directed to the corresponding author.

## Ethics statement

Ethical review and approval were not required for this study in accordance with the local legislation and institutional requirements, because it is retrospective in nature and included clinical cases; hence, it is exempt from IACUC requirements. Written informed consent for participation was not obtained from the owners because the study is retrospective in nature and hence, it is exempt from written informed consent.

## Author contributions

SM: Data curation, Validation, Visualization, Writing—original draft, Writing—review & editing. EA: Investigation, Validation, Visualization, Writing—review & editing. SB: Investigation, Validation, Visualization, Writing—review & editing. MK: Data curation, Formal analysis, Investigation, Validation, Visualization, Writing—review & editing. MG: Conceptualization, Data curation, Funding acquisition, Investigation, Validation, Visualization, Writing—review & editing.

## Supplementary material

Supplementary Table 1 Complete population data ordered by patient number: patient number, number of consultations, breed, sex (F, female; M, male), neutering status (I, intact; N, neutered), bodyweight (Kg), age (months), DV symphyseal mobility score, LM symphyseal mobility score, symphyseal radiographic appearance (D, divergent; F, fused; P, parallel; blackened cells: excluded from statistical analysis), and skull morphology (B, brachycephalic; NB, non- brachycephalic).

## References

[1] Evans HE De Lahunta A. The skeleton. In:Miller ME Evans HE, editor Miller's Anatomy of the Dog, 4th Edn. St. Louis, MI: Saunders/Elseviers (2013), p. 80–159.

[2] Scapino RP. The third joint of the canine jaw. J Morphol. (1965) 116:23–50. 10.1002/jmor.1051160103 14294954

[3] Scapino RP. Morphological investigation into functions of the jaw symphysis in carnivorans. J Morphol. (1981) 167:339–75. 10.1002/jmor.1051670308 7241602

[4] Beecher RM. Function and fusion at the mandibular symphysis. Am J Physical Anthropology. (1977) 47:325–35. 10.1002/ajpa.1330470213 410309

[5] Beecher RM. Functional significance of the mandibular symphysis. J Morphology. (1979) 159:117–30. 10.1002/jmor.1051590109 423251

[6] Scott JE Hogue AS Ravosa MJ. The adaptive significance of mandibular symphysial fusion in mammals. J Evol Biol. (2012) 25:661–73. 10.1111/j.1420-9101.2012.02457.x 22268953

[7] Scott JE Lack JB Ravosa MJ. On the reversibility of mandibular symphysial fusion. Evolution. (2012) 66:2940–52. 10.1111/j.1558-5646.2012.01639.x 22946814

[8] Evans HE De Lahunta A. Arthrology. In:Miller ME Evans HE, editor Miller's Anatomy of the Dog, 4th Edn. St. Louis, MI: Saunders/Elseviers (2013), p. 158–184.

[9] Greaves WS. A. functional consequence of an ossified mandibular symphysis. Am J Phys Anthropol. (1988) 77:53–6. 10.1002/ajpa.1330770109 3189523

[10] Lieberman DE Crompton AW. Why fuse the mandibular symphysis? A comparative analysis. Am J Phys Anthropol. (2000) 112:517–40. 10.1002/1096-8644(200008)112:4&lt;517::AID-AJPA7&gt;3.0.CO;2-4 10918127

[11] Ravosa MJ Hylander WL. Functional significance of an ossified mandibular symphysis: a reply. Am J Physical Anthropology. (1993) 90:509–14. 10.1002/ajpa.1330900412 8476008

[12] Ravosa MJ Kunwar R Stock SR Stack MS. Pushing the limit: masticatory stress and adaptive plasticity in mammalian craniomandibular joints. J Exp Biol. (2007) 210:628–41. 10.1242/jeb.02683 17267649

[13] Ravosa MJ Ning J Costley DB Daniel AN Stock SR Stack MS. Masticatory biomechanics and masseter fiber-type plasticity. J Muscoloskelet Neuronal Interact. (2010) 10:46–55. 20190379

[14] Gawor J. Dental radiographic interpretation. In:Niemec BA Gawor J Jekl V, editors, Practical Veterinary Dental Radiography. Boca Raton, FL: CRC Press, Taylor & Francis Group, (2017), p. 99–220.

[15] Gawor J. Radiography of the temporomandibular joint and mandibular symphysis. In:Niemec BA Gawor J Jekl V, editors, Practical Veterinary Dental Radiography. Boca Raton, FL: CRC Press, Taylor & Francis Group, (2017), p. 249–70.

[16] Niemec BA. Oral radiology and imaging. In:Lobprise HB Dodd JR, editors, Wiggs's Veterinary Dentistry: Principles and Practice, 2nd Edn. Hoboken, NJ, USA: Wiley & Sons Inc (2019), p.41–62.

[17] Gioso MA Carvalho VG. Oral anatomy of the dog and cat in veterinary dentistry practice. Vet Clin North Am Small Anim Pract. (2005) 35:763–80. 10.1016/j.cvsm.2004.10.003 15979512

[18] Meola SD. Brachycephalic airway syndrome. Top Companion Anim Med. (2013) 28:91–6. 10.1053/j.tcam.2013.06.004 24182996

[19] Dupré G Heidenreich D. Brachycephalic syndrome. Vet Clin North Am Small Anim Pract. (2016) 46:691–707. 10.1016/j.cvsm.2016.02.002 27012936

[20] Kyllar M Paral V Pyszko M Doskarova B. Facial pillars in dogs: an anatomical study. Anat Sci Int. (2017) 92:343–51. 10.1007/s12565-016-0338-x 27015686

[21] Downing F Gibson S. Anaesthesia of brachycephalic dogs. J Small Anim Pract. (2018) 59:725–33. 10.1111/jsap.12948 30374971

[22] Kim YJ Lee N Yu J Lee H An G Bang S. Three-dimensional volumetric magnetic resonance imaging (MRI) analysis of the soft palate and nasopharynx in brachycephalic and non-brachycephalic dog breeds. J Vet Med Sci. (2019) 81:113–9. 10.1292/jvms.17-0711 30518706 PMC6361641

[23] Ekenstedt KJ Crosse KR Risselada M. Canine brachycephaly: anatomy, pathology, genetics and welfare. J Comp Pathol. (2020) 176:109–15. 10.1016/j.jcpa.2020.02.008 32359622 PMC7380493

[24] Krainer D Dupré G. Brachycephalic obstructive airway syndrome. Vet Clin North Am Small Anim Pract. (2022) 52:749–80. 10.1016/j.cvsm.2022.01.013 35379494

[25] American Veterinary Dental College,. Nomenclature. Periodontal Anatomy Disease. Stages of Periodontal Disease. (2023). Available online at: https://avdc.org/avdc-nomenclature/ (accessed June 25, 2023).

[26] Evans HE De Lahunta A. Muscles of the head. In:Miller ME Evans HE, editors Miller's Anatomy of the Dog, 4th Edn. St. Louis, MI: Saunders/Elseviers (2013), p. 191–210.

[27] Schoenebeck JJ Ostrander EA. The genetics of canine skull shape variation. Genetics. (2013) 193:317–25. 10.1534/genetics.112.145284 23396475 PMC3567726

[28] Buzek A Serwańska-Leja K Zaworska-Zakrzewska A Kasprowicz-Potocka M. The shape of the nasal cavity and adaptations to sniffing in the dog (*Canis familiaris*) compared to other domesticated mammals: a review article. Animals. (2022) 12:517. 10.3390/ani12040517 35203225 PMC8868339

[29] Ellis JL Thomason JJ Kebreab E Zubair K France J. Cranial dimensions and forces of biting in the domestic dog. J Anat. (2009) 214:362–73. 10.1111/j.1469-7580.2008.01042.x 19245503 PMC2673787

[30] Kim SE Arzi B Garcia TC Verstraete FJM. Bite forces and their measurement in dogs and cats. Front Vet Sci. (2018) 13:76. 10.3389/fvets.2018.00076 29755988 PMC5932386

[31] Brassard C Merlin M Guintard C Monchâtre-Leroy E Barrat J Bausmayer N. Bite force and its relationship to jaw shape in domestic dogs. J Exp Biol. (2020) 17:jeb224352. 10.1242/jeb.224352 32587065

[32] Legendre L. Maxillofacial fracture repairs. Vet Clin North Am Small Anim Pract. (2005) 35:985–1008. 10.1016/j.cvsm.2005.03.003 15979522

[33] Zacher AM Marretta SM. Oral and maxillofacial surgery in dogs and cats. Vet Clin North Am Small Anim Pract. (2013) 43:609–49. 10.1016/j.cvsm.2013.02.010 23643024

[34] De Paolo MH Arzi B Pollard RE Kass PH Verstraete FJM. Craniomaxillofacial trauma in dogs — Part I: fracture location, morphology and etiology. Front Vet Sci. (2020) 7:241. 10.3389/fvets.2020.00241 32411743 PMC7199291

[35] De Paolo MH Arzi B Pollard RE Kass PH Verstraete FJM. Craniomaxillofacial trauma in dogs —Part II: association between fracture location, morphology and etiology. Front Vet Sci. (2020) 7:242. 10.3389/fvets.2020.00242 32478108 PMC7242568

[36] Stewart WC Baker GJ Lee R. Temporomandibular subluxation in the dog: a case report. J Small Anim Pract. (1975) 16:345–549. 10.1111/j.1748-5827.1975.tb05752.x 1152443

[37] Robins G Grandage J. Temporomandibular joint dysplasia and open-mouth jaw locking in the dog. J Am Vet Med Assoc. (1977) 171:1072–6. 591420

[38] Thomas RE. Temporo-mandibular joint dysplasia and open-mouth jaw locking in a Bassett Hound: a case report. J Small Anim Pract. (1979) 20:697–701. 10.1111/j.1748-5827.1979.tb06684.x 547116

[39] Johnson KA. Temporomandibular joint dysplasia in an Irish Setter. J Small Anim Pract. (1979) 20:209–18. 10.1111/j.1748-5827.1979.tb06708.x 439872

[40] Lantz GC Cantwell HD. Intermittent open-mouth lower jaw locking in five dogs. J Am Vet Med Assoc. (1986) 188:1403–5. 3744967

[41] Hzewinkel HAW Koole R Voorhout G. Mandibular coronoid process displacement: signs, causes, treatment. Vet Comp Orthop Traumatol. (1993) 6:29–35. 10.1055/s-0038-1633143

[42] Ryan J Fraga-Manteiga E Schwarz T Clements DN. Unilateral synostosis of the zygomaticotemporal suture associated with mandibular coronoid process impingement in a dog. Vet Comp Orthop Traumatol. (2013) 26:421–4. 10.3415/VCOT-12-12-0157 23709015

[43] Arzi B Cissell DD Verstraete FJ Kass PH DuRaine GD Athanasiou KA. Computed tomographic findings in dogs and cats with temporomandibular joint disorders: 58 cases (2006-2011). J Am Vet Med Assoc. (2013) 242:69–75. 10.2460/javma.242.1.69 23234284 PMC3747040

